# Identification of a Flavivirus Sequence in a Marine Arthropod

**DOI:** 10.1371/journal.pone.0146037

**Published:** 2015-12-30

**Authors:** Michael J. Conway

**Affiliations:** Foundational Sciences, Central Michigan University, College of Medicine, Mt. Pleasant, Michigan, 48859, United States of America; Defence Research Laboratory, INDIA

## Abstract

Phylogenetic analysis has yet to uncover the early origins of flaviviruses. In this study, I mined a database of expressed sequence tags in order to discover novel flavivirus sequences. Flavivirus sequences were identified in a pool of mRNA extracted from the sea spider *Endeis spinosa* (Pycnogonida, Pantopoda). Reconstruction of the translated sequences and BLAST analysis matched the sequence to the flavivirus NS5 gene. Additional sequences corresponding to envelope and the NS5 MTase domain were also identified. Phylogenetic analysis of homologous NS5 sequences revealed that *Endeis spinosa* NS5 (ESNS5) is likely related to classical insect-specific flaviviruses. It is unclear if ESNS5 represents genetic material from an active viral infection or an integrated viral genome. These data raise the possibility that classical insect-specific flaviviruses and perhaps medically relevant flaviviruses, evolved from progenitors that infected marine arthropods.

## Introduction

Viruses in the genus *Flavivirus* share a common genomic organization and certain antigenic relationships; however, they can be divided into distinct phylogenetic groups [1]. Important groups include the mosquito-borne and tick-borne flaviviruses, flaviviruses with no known arthropod vector, dual host associated insect-specific flaviviruses (dhISFVs), and classical insect-specific flaviviruses (cISFVs) [2, 3]. Flaviviruses are clustered into phylogenetic groups likely due to selective pressures imposed on the viruses by their unique transmission cycles and ecologies [4–8]. Many mosquito and tick-borne flaviviruses are medically relevant in that they cause hemorrhagic and encephalitic disease in humans. It is unclear if vector co-infection with insect-specific flaviviruses impacts disease transmission or pathogenesis in nature [2, 9, 10].

Phylogenetic analysis of flaviviruses has been reported using individual genes and whole genomes and trees have been built using both amino acid and nucleotide sequences [1, 11–16]. These studies have yet to uncover the early origins of flaviviruses [11, 12]. Molecular clock studies have been unreliable due to dramatic changes in the nucleotide substitution rate over time and differences in the mutation rate between individual flaviviruses [16, 17]. Further, our current database of genetic material may only represent the tips of the evolutionary tree [12, 17].

In order to increase the pool of flavivirus sequences available for phylogenetic analysis, I mined an expressed sequence tag (est) database against a number of flavivirus genomes. Data mining identified flavivirus sequences in a library of sea spider *Endeis spinosa* (Pycnogonida, Pantopoda) cDNAs. Reconstruction of the translated sequences and BLAST analysis identified the protein as flavivirus NS5. Additional sequences corresponding to envelope and the NS5 MTase domain were also identified. Flavivirus sequences have been previously identified in the genomes of disease vectors, suggesting that integration of flaviviral genomes into the germline of arthropod hosts is a common event. It is unclear if ESNS5 represents genetic material from an active viral infection or an integrated viral genome. Phylogenetic analysis indicated that *Endeis spinosa* NS5 (ESNS5) shares a common ancestor that predates the evolution of classical insect-specific flaviviruses. These data suggest that classical insect-specific flaviviruses, and perhaps medically relevant flaviviruses, evolved from progenitors who first infected marine arthropods.

## Materials and Methods

### RNA extraction, library construction, and sequencing

RNA extraction, library construction, and sequencing was performed previously by Meusemann and Burmester et al., and uploaded onto the GenBank database as unpublished data on the expressed sequence tag (est) database. Briefly, *Endeis spinosa* were collected with the help of scientists from the Istituto di Scienze Marine, Venice (Italy). Animals were shock-frozen and grinded in liquid nitrogen. Total RNA was extracted as described by Holmes and Bonner (1973) and was further purified using the NucleoSpin RNA II kit (Macherey- Nagel, Dueren, Germany) including a DNase digest. Poly(A)+ RNA was enriched from the total RNA using Dynabeads Oligo(dT)_25_ (Invitrogen, Carlsbad, USA). ESTs were sequenced within the project "Molecular Phylogeny of the Arthropoda and the 'Ecdysozoa' Hypothesis", University of Hamburg, founded by the DFG priority program SPP 1174 "Deep Metazoan Phylogeny". Library construction and sequencing were performed at the MPI for Molecular Genetics, Berlin, Germany. The same research group constructed and uploaded cDNA libraries onto the est database representing the following terrestrial and marine arthropods: *Anurida maritima*, *Acerentomon franzi*, *Campodea fragilis*, *Lepismachilis y-signata*, *Speleonectes cf*. *tulumensis*, *Archispirostreptus gigas*, *Limulus polyphemus*, *Peripatopsis sedgwicki*, *Tigriopus californicus*, *Pollicipes pollicipes*, and *Triops cancriformis*.

### Genetic analysis

Translated nucleotide BLAST (tblastn) database searches were performed by searching for flavivirus sequences in the expressed sequence tag (est) database. 100 cDNA clones were identified and accession numbers are available in S1 Table. ESNS5 and NS5 amino acid sequences for nearly all flaviviruses were aligned using MUSCLE multiple sequence alignment [18] and Gblocks 0.91b software [19]. A stringent selection that did not allow contiguous nonconserved positions was chosen. Aligned sequences were uploaded into MEGA6 software in FASTA format [20]. Evolutionary histories were inferred by analyzing aligned amino acid sequences by the Maximum Likelihood method based on the JTT matrix-based models. The robustness of the resulting groupings was tested by 1,000 bootstrap replications. The tree with the highest log likelihood was shown. The percentage of trees in which the associated taxa clustered together was shown next to the branches. Initial trees for the heuristic search were obtained by applying the Neighbor-Joining method to a matrix of pairwise distances estimated using either the JTT approach. Trees were drawn to scale, with branch lengths measured in the number of substitutions per site. All positions containing gaps and missing data were eliminated.

## Results

### Bioinformatic detection and reconstruction of ESNS5

**Fig 1:**
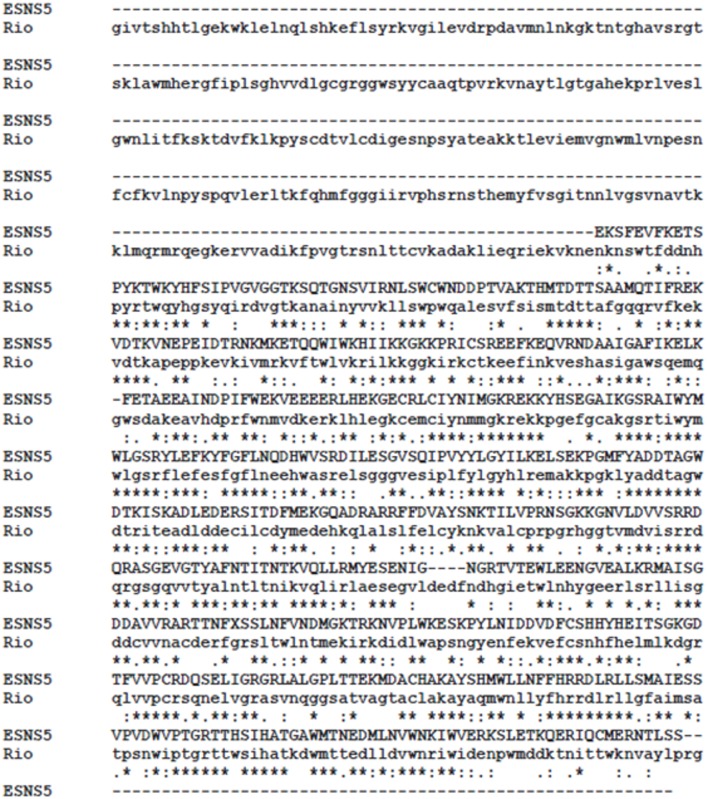
Alignment of ESNS5 and Rio Bravo virus NS5 RdRp. Amino acid sequence alignment was performed using reconstructed ESNS5 and Rio Bravo virus NS5 RdRp (Rio) using Clustal Omega. Stars represent perfect homology, colons represent partial homology, and periods represent weak homology. Dashes represent deleted sequence.

To identify novel flavivirus sequences, translated nucleotide BLAST (tblastn) database searches were performed by searching for flavivirus sequences in the expressed sequence tag (est) database. Whole flavivirus polyproteins were used to search the database. Tblastn analysis using the Sepik virus polyprotein identified multiple cDNA clones constructed from mRNA isolated from the sea spider *Endeis spinosa*. The top 10 clones are included in Table 1. To verify the presence of flavivirus sequences in the est database, nucleotide BLAST analysis was performed using the complete Sepik virus genome. Searches were performed with all three program selections: (1) highly similar sequences (megablast), (2) more dissimilar sequences (discontiguous megablast), and (3) somewhat similar sequences (blastn). Using this strategy, 76 cDNA clones were identified that matched to the Sepik virus genome. The top 10 clones are included in Table 2. Each of the cDNA clones identified from both search strategies were translated and the protein was reconstructed by amino acid alignment (Fig 1). Protein BLAST analysis suggested that this protein, which we call *Endeis spinosa* NS5 (ESNS5), is most related to “no known vector” and tick-borne flaviviruses (Table 3). ESNS5 represented a fragment of a NS5 RNA dependent RNA polymerase (RdRp) domain. Protein alignment with Rio Bravo virus (RBV) NS5 RdRp showed significant internal homology, but deletions at the N- and C-termini (Fig 1). A stop codon was evident in ESNS5, confirming that a small deletion is present at the C-terminus. An additional cDNA mining attempt was performed with tblastn by searching the expressed sequence tags (est) database for sequences that match the RBV polyprotein. Using this search strategy, additional clones that matched the flavivirus envelope protein and NS5 MTase domains were identified (S1 Table).

**Table 1:** Top 10 tblastn hits of Sepik virus polyprotein against the expressed sequence tags (est) database.

Best hit	Accession number	E-value
*Endeis spinosa* cDNA	GI:283511872	6e-71
*Endeis spinosa* cDNA	GI:283498993	1e-70
*Endeis spinosa* cDNA	GI:283545672	2e-68
*Endeis spinosa* cDNA	GI:283545171	2e-67
*Endeis spinosa* cDNA	GI:283541209	1e-66
*Endeis spinosa* cDNA	GI:283513154	2e-66
*Endeis spinosa* cDNA	GI:283534952	6e-66
*Endeis spinosa* cDNA	GI:283535103	7e-66
*Endeis spinosa* cDNA	GI:283545130	9e-66
*Endeis spinosa* cDNA	GI:283545705	9e-66

**Table 2:** Top 10 nucleotide BLAST hits of Sepik virus genome against the expressed sequence tags (est) database.

Best hit	Accession number	E-value
*Endeis spinosa* cDNA	GI:283541362	8e-16
*Endeis spinosa* cDNA	GI:283538836	8e-16
*Endeis spinosa* cDNA	GI:283511744	8e-16
*Endeis spinosa* cDNA	GI:283498993	8e-16
*Endeis spinosa* cDNA	GI:283545705	4e-14
*Endeis spinosa* cDNA	GI:283545672	4e-14
*Endeis spinosa* cDNA	GI:283545171	4e-14
*Endeis spinosa* cDNA	GI:283545118	4e-14
*Endeis spinosa* cDNA	GI:283541204	4e-14
*Endeis spinosa* cDNA	GI:283535170	4e-14

**Table 3:** Top 10 protein BLAST hits of reconstructed ESNS5 against the non-redundant protein sequence (nr) database.

Best hit	Accession number	E-value
Rio Bravo virus NS5	GI:27735371	3e-180
Montana myotis leukoencephalitis virus NS5	GI:27669998	2e-178
Deer tick virus NS5	GI:164504431	2e-178
Tick-borne encephalitis virus NS5	GI:27596786	3e-178
Alkhumra hemorrhagic fever virus NS5	GI:27545515	1e-177
Modoc virus NS5	GI:25121731	7e-177
Powassan virus NS5	GI:16945860	9e-176
Langat virus NS5	GI:25121534	1e-175
Apoi virus NS5	GI:27697405	3e-175
Dengue virus type 1 NS5	GI:25014069	5e-174

The *Endeis spinosa* cDNA library was performed by RNA extraction and purification using a NucleoSpin RNA II kit. A DNase digest was performed to eliminate genomic DNA contamination. Poly(A)+ RNA was enriched from the total RNA using Dynabeads Oligo(dT)_25_ and clones were sequenced by Sanger sequencing. Interestingly, no 3’ poly(A) tails were identified in any of the ESNS5 clones. Instead, internal tracts of poly(A) sequences were observed. The majority of clones contained the following internal poly(A) tract: “aaaaaaggaaa”. Additional internal poly(A) tracts were also identified in ESNS5 clones, suggesting that the flavivirus sequences may not have derived from an endogenous gene. G+C content was calculated for *Endeis spinosa* envelope (39.9%) and NS5 (41.1%) sequences, *Endeis spinosa* actin (GenBank accession number FN213165) (46.8%), histone H3 (GenBank accession number FJ862879) (47.5%), and 18S ribosomal RNA (GenBank accession number FJ862848) (51.2%), and the Rio Bravo virus genome (GenBank accession number AF144692) (43.2%). The flavivirus sequences had noticeably lower G+C content than the *Endeis spinosa* genes and were more similar to the G+C content of Rio Bravo virus.

The same laboratory that uploaded the *Endeis spinosa* cDNA library constructed and uploaded cDNA libraries for 11 other terrestrial and marine arthropods: *Anurida maritima*, *Acerentomon franzi*, *Campodea fragilis*, *Lepismachilis y-signata*, *Speleonectes cf*. *tulumensis*, *Archispirostreptus gigas*, *Limulus polyphemus*, *Peripatopsis sedgwicki*, *Tigriopus californicus*, *Pollicipes pollicipes*, and *Triops cancriformis*. These libraries equal 62,739 cDNA clones. Flavivirus sequences were not identified in any of the above cDNA libraries while using tblastn to search the est database for sequences that match the RBV polyprotein. Laboratory contamination was likely not responsible for the presence of flavivirus sequences in the *Endeis spinosa* library.

### The phylogenetic position of ESNS5

**Fig 2:**
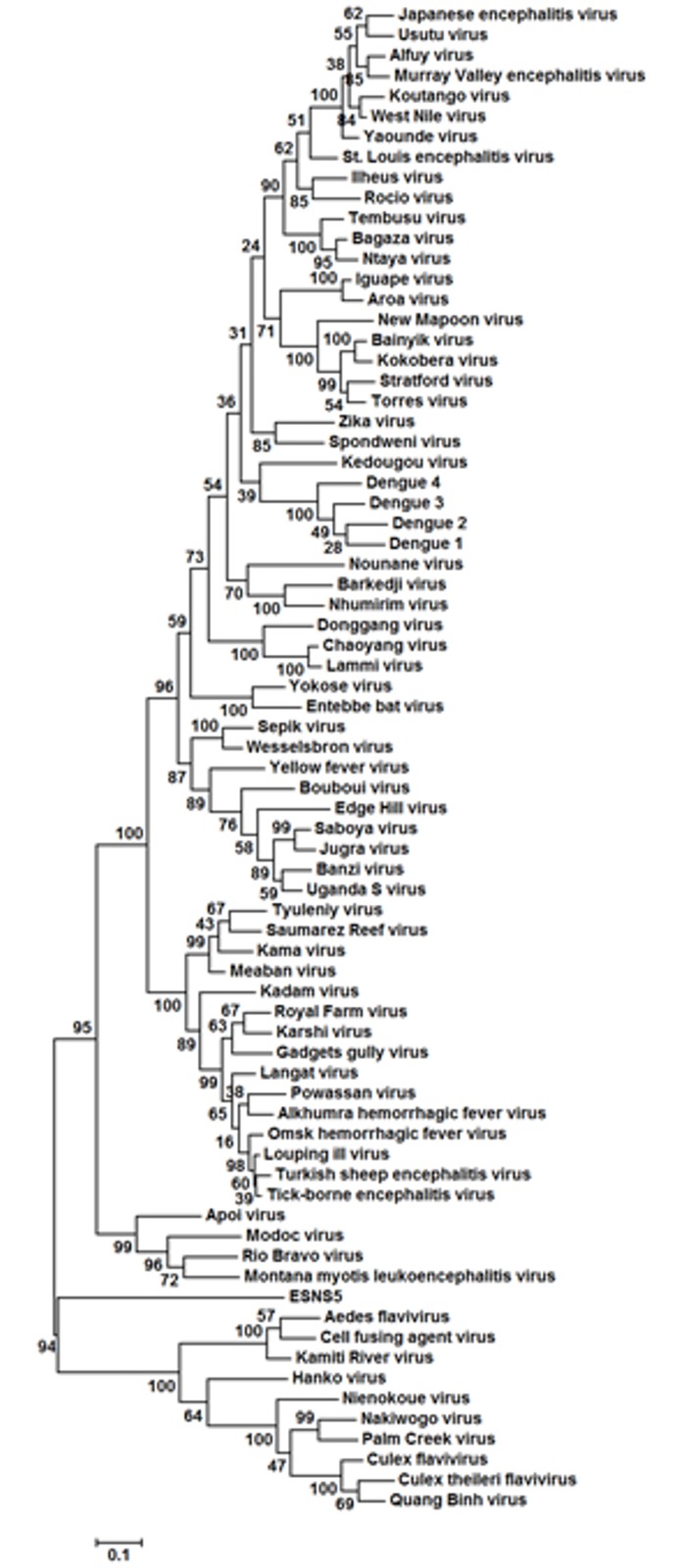
Phylogenetic position of ESNS5. The evolutionary history of ESNS5 and almost all known flaviviruses was inferred by generating homologous NS5 amino acid sequences using MUSCLE multiple sequence alignment and Gblocks 0.91b software, followed by the Maximum Likelihood methods based on the JTT matrix-based models. The tree with the highest log likelihood (-27149.5525) is shown. The percentage of trees in which the associated taxa clustered together is shown next to the branches. Initial trees for the heuristic search were obtained by applying the Neighbor-Joining method to a matrix of pairwise distances estimated using a JTT model. The tree is drawn to scale, with branch lengths measured in the number of substitutions per site. The analysis involved 74 sequences. All positions containing gaps and missing data were eliminated. There were a total of 496 positions in the final dataset. Evolutionary analysis was conducted in MEGA6.

In order to determine the phylogenetic relationship of ESNS5 with extant flaviviruses, a phylogenetic tree was constructed with homologous NS5 amino acid sequences from almost all known flaviviruses (Fig 2). The Maximum Likelihood method was employed using a JTT matrix-based model for amino acid alignments. Initial trees were obtained by applying the Neighbor-Joining method. The phylogeny supports that ESNS5 is related to cISFVs, and raises the possibility that cISFVs may have derived from a marine arthropod.

## Discussion

*Endeis spinosa* is a member of the Pycnogonida, which are commonly known as sea spiders [21, 22]. These marine arthropods are ubiquitous and various species are found distributed around the world [23]. Most species have a body only mm in length; however, giant sea spiders have been isolated in Antarctic waters [23]. Genetic evidence suggests that Pycnogonida may be an ancient sister group to all living arthropods—a history that spans hundreds of millions of years [24].

It is unclear at this point if ESNS5 represents an active infection, a very old integration event, or an integration event from an extant but previously unidentified flavivirus. The cDNA library was constructed using Dynabeads Oligo(dT)_25_, which purify mRNA with high sensitivity and specificity. Viral RNA contamination from infected sea spider tissue may have occurred through binding of internal poly(A) tracts to the Dynabeads, which were identified in many of the ESNS5 clones. RNA with internal poly(A) tracts can contaminate mRNA during enrichment with oligo-dT, and this would be more likely if the RNA had a high copy number such as a replicating viral genome [25, 26]. 3’ poly(A) tracts are only found in prototype tick-borne encephalitis virus strain Neudoerfl [27, 28]. Further, it is unlikely that ESNS5 represents laboratory contamination since flavivirus sequences were not identified in 11 other cDNA libraries that were constructed, sequenced, and uploaded by the same laboratory. G+C content was also lower than *Endeis spinosa* genes and was closer to the G+C content in Rio Bravo virus.

It is theoretically possible that ESNS5 and its related envelope and MTase sequences represent a genome-wide integration event or multiple integration events. Genetic studies have estimated a long history of flaviviral infections in mosquitoes, and this analysis extends into the genomes of key disease vectors [13, 29]. PCR techniques and whole genomic sequencing of *Ae*. *albopictus* and *Ae*. *aegypti* revealed fragments of ancestral viral infections throughout both genomes [29, 30]. Approximately two-thirds of a flavivirus-like genome were identified in *Ae*. *albopictus* integrated as a single open reading frame spanning NS1-NS4A [29]. *Ae*. *albopictus* and *Ae*. *aegypti* share a stretch of sequence that resembles flavivirus NS1, but they do not appear to be phylogenetically related [29]. Additional unique sequences, including a stretch of sequence resembling NS5 were identified in *Ae*. *aegypti* (AENS5) but not in *Ae*. *albopictus* [29].

This study identified a flavivirus sequence in a cDNA library from a marine arthropod and showed a phylogenetic relationship with classical insect-specific flaviviruses. Additional research is necessary to determine if marine arthropods can be and are currently infected with flaviviruses.

## Supporting Information

S1 Table GenBank accession numbers of sequences analyzed in this study. (DOCX)
