# Supplementary material for: Identification of a Flavivirus Sequence in a Marine Arthropod
Source: PLoS One. 2015 Dec 30;10(12):e0146037. doi: 10.1371/journal.pone.0146037 (PMC4699914; doi:10.1371/journal.pone.0146037)
Supplement: S1 Table — (DOCX) [file pone.0146037.s001.docx]

S1 Table. GenBank accession numbers of sequences analyzed in this study

| Sequences | Accession |
| --- | --- |
| *Endeis spinosa* RdRp | FN215188 |
| *Endeis spinosa* RdRp | FN214012 |
| *Endeis spinosa* RdRp | FN211565 |
| *Endeis spinosa* RdRp | FN212778 |
| *Endeis spinosa* RdRp | FN212227 |
| *Endeis spinosa* RdRp | FN213800 |
| *Endeis spinosa* RdRp | FN215195 |
| *Endeis spinosa* RdRp | FN212605 |
| *Endeis spinosa* RdRp | FN214648 |
| *Endeis spinosa* RdRp | FN213895 |
| *Endeis spinosa* RdRp | FN212467 |
| *Endeis spinosa* RdRp | FN215130 |
| *Endeis spinosa* RdRp | FN214616 |
| *Endeis spinosa* RdRp | FN214720 |
| *Endeis spinosa* RdRp | FN213123 |
| *Endeis spinosa* RdRp | FN212576 |
| *Endeis spinosa* RdRp | FN213365 |
| *Endeis spinosa* RdRp | FN214646 |
| *Endeis spinosa* RdRp | FN213631 |
| *Endeis spinosa* RdRp | FN213735 |
| *Endeis spinosa* RdRp | FN214161 |
| *Endeis spinosa* RdRp | FN212311 |
| *Endeis spinosa* RdRp | FN212387 |
| *Endeis spinosa* RdRp | FN214655 |
| *Endeis spinosa* RdRp | FN213384 |
| *Endeis spinosa* RdRp | FN213348 |
| *Endeis spinosa* RdRp | FN214412 |
| *Endeis spinosa* RdRp | FN211930 |
| *Endeis spinosa* RdRp | FN212194 |
| *Endeis spinosa* RdRp | FN211758 |
| *Endeis spinosa* RdRp | FN211699 |
| *Endeis spinosa* RdRp | FN215076 |
| *Endeis spinosa* RdRp | FN212640 |
| *Endeis spinosa* RdRp | FN212974 |
| *Endeis spinosa* RdRp | FN214209 |
| *Endeis spinosa* RdRp | FN213760 |
| *Endeis spinosa* RdRp | FN211414 |
| *Endeis spinosa* RdRp | FN213640 |
| *Endeis spinosa* RdRp | FN213006 |
| *Endeis spinosa* RdRp | FN213613 |
| *Endeis spinosa* RdRp | FN214523 |
| *Endeis spinosa* RdRp | FN215147 |
| *Endeis spinosa* RdRp | FN213870 |
| *Endeis spinosa* RdRp | FN213594 |
| *Endeis spinosa* RdRp | FN214498 |
| *Endeis spinosa* RdRp | FN211694 |
| *Endeis spinosa* RdRp | FN213821 |
| *Endeis spinosa* RdRp | FN214996 |
| *Endeis spinosa* RdRp | FN212550 |
| *Endeis spinosa* RdRp | FN214650 |
| *Endeis spinosa* RdRp | FN211774 |
| *Endeis spinosa* RdRp | FN213939 |
| *Endeis spinosa* RdRp | FN215293 |
| *Endeis spinosa* RdRp | FN213503 |
| *Endeis spinosa* RdRp | FN214341 |
| *Endeis spinosa* RdRp | FN213577 |
| *Endeis spinosa* RdRp | FN212422 |
| *Endeis spinosa* RdRp | FN212000 |
| *Endeis spinosa* RdRp | FN214240 |
| *Endeis spinosa* RdRp | FN213316 |
| *Endeis spinosa* RdRp | FN212823 |
| *Endeis spinosa* RdRp | FN212203 |
| *Endeis spinosa* RdRp | FN211990 |
| *Endeis spinosa* RdRp | FN213411 |
| *Endeis spinosa* RdRp | FN215135 |
| *Endeis spinosa* RdRp | FN213150 |
| *Endeis spinosa* RdRp | FN213996 |
| *Endeis spinosa* RdRp | FN212147 |
| *Endeis spinosa* RdRp | FN213823 |
| *Endeis spinosa* RdRp | FN213157 |
| *Endeis spinosa* RdRp | FN214177 |
| *Endeis spinosa* RdRp | FN213387 |
| *Endeis spinosa* RdRp | FN213880 |
| *Endeis spinosa* RdRp | FN213144 |
| *Endeis spinosa* RdRp | FN211478 |
| *Endeis spinosa* RdRp | FN215145 |
| *Endeis spinosa* RdRp | FN211540 |
| *Endeis spinosa* RdRp | FN213008 |
| *Endeis spinosa* RdRp | FN213803 |
| *Endeis spinosa* MTase | FN211958 |
| *Endeis spinosa* RdRp | FN212906 |
| *Endeis spinosa* MTase | FN213818 |
| *Endeis spinosa* MTase | FN212346 |
| *Endeis spinosa* MTase | FN213035 |
| *Endeis spinosa* RdRp | FN212981 |
| *Endeis spinosa* MTase | FN214758 |
| *Endeis spinosa* RdRp | FN211695 |
| *Endeis spinosa* MTase | FN213167 |
| *Endeis spinosa* MTase | FN211610 |
| *Endeis spinosa* RdRp | FN215137 |
| *Endeis spinosa* MTase | FN212997 |
| *Endeis spinosa* MTase | FN213276 |
| *Endeis spinosa* MTase | FN211621 |
| *Endeis spinosa* MTase | FN213258 |
| *Endeis spinosa* RdRp | FN213492 |
| *Endeis spinosa* RdRp | FN214081 |
| *Endeis spinosa* RdRp | FN213080 |
| *Endeis spinosa* RdRp | FN214726 |
| *Endeis spinosa* MTase | FN211464 |
| *Endeis spinosa* envelope | FN213658 |
